# Comparative evaluation of propolis and *Persea americana* creams for epidermal wound healing in rats

**DOI:** 10.1038/s41598-025-17070-6

**Published:** 2025-08-27

**Authors:** Yasmin M. Abd El-Aziz, Yara Allam, Mariam Abdelsed, Sama Gabr, Mariam Mohamed, Jomana Samir, Donia Elgamil, Farah Farahat, Monica Gerges, Menna Hany, Ali H. Abu Almaaty

**Affiliations:** Department of Zoology, Faculty of Science, Port Said University, Port Said, 42526 Egypt

**Keywords:** GC–MS, Histochemistry, Histopathology, *Persea americana*, Propolis, Wound, Cell biology, Physiology, Zoology, Health care, Medical research

## Abstract

Wound healing is a complex biological process that benefits greatly from effective topical treatments. This study compares the healing potential of two natural formulations: propolis cream and *Persea americana* cream. Twelve albino rats were divided into four groups: a negative control group with no injury, a positive control group with injury but no treatment, a group treated with propolis cream post-injury, and a group treated with the *Persea americana* cream post-injury, with three rats in each group. All wounds were sterilized daily using Betadine. The experiment lasted for 15 days. Results indicated that the *Persea americana* cream demonstrated superior therapeutic effects compared to the propolis cream. Blood samples were collected to assess the systemic influence of the treatments on the wound healing process, under appropriate preservation conditions. Skin specimens were then taken for histopathological examination using Hematoxylin & Eosin and Masson’s trichrome staining to observe biochemical changes in wound tissues relative to healthy skin. Additionally, GC/MS analysis was conducted to identify and compare the bioactive compounds present in both creams. The analysis revealed that the *Persea americana* cream contained a higher concentration of bioactive compounds, which likely contributed to the enhanced healing observed. Overall, the formulations were designed to utilize the bioactive components to accelerate wound repair effectively.

## Introduction

Wound healing is a dynamic biological process involving a coordinated series of events that restore tissue integrity following injury. This process encompasses four overlapping phases: hemostasis, inflammation, proliferation, and maturation. Initially, blood vessels constrict and platelets aggregate to form a fibrin clot that prevents excessive bleeding. The inflammatory phase follows, characterized by infiltration of neutrophils and macrophages, which release cytokines such as IL-1 and IL-6 to combat infection and initiate tissue repair. Subsequently, fibroblasts migrate into the wound area, synthesizing key extracellular matrix components like collagen and hyaluronic acid, while keratinocytes proliferate and differentiate to re-establish the epidermal barrier. The final maturation phase involves remodeling of the extracellular matrix and formation of scar tissue, a process that may extend over several months to years^1^.

Despite the availability of various wound care products, many remain inaccessible or present side effects, prompting the exploration of natural remedies with therapeutic potential^2^. Among these, Propolis and *Persea americana* (avocado) have demonstrated promising biological activities conducive to wound healing.

Propolis, a resinous substance collected by bees from plant sources, is renowned for its antimicrobial, anti-inflammatory, and antioxidant properties^2–4^. It has been traditionally applied in treating burns, infected wounds, and other skin injuries. Chemical studies have identified an array of bioactive constituents in propolis, including flavonoids such as pinocembrin and galangin, phenolic acids like caffeic acid and CAPE, as well as various terpenes. These compounds exert synergistic effects, neutralizing oxidative stress, modulating inflammatory responses, promoting collagen synthesis, and enhancing fibroblast activity all crucial factors in effective wound repair^5–7^.

Similarly, *Persea americana* is rich in phytochemicals that facilitate skin regeneration. GC/MS analysis of the *Persea americana* cream formulated from avocado reveals significant compounds such as 9,12-octadecenoic acid (Z,Z), which improves skin hydration, accelerates wound healing, and stimulates growth factors like VEGF and IL-1β. Other constituents including oleic acid derivatives, linolenic acid esters, and n-hexadecanoic acid contribute antimicrobial, anti-inflammatory, and antioxidant effects that protect the wound site and support tissue restoration^8–16^.

Considering the multifaceted biological activities of both propolis and avocado extracts, this study aims to comparatively evaluate their efficacy in promoting epidermal wound healing in an albino rat model, integrating clinical observations, histopathological evaluation, and chemical profiling to elucidate their healing mechanisms.

## Materials and methods

### Bioactive compound creams

#### Commercial propolis cream

**Fig. 1:**
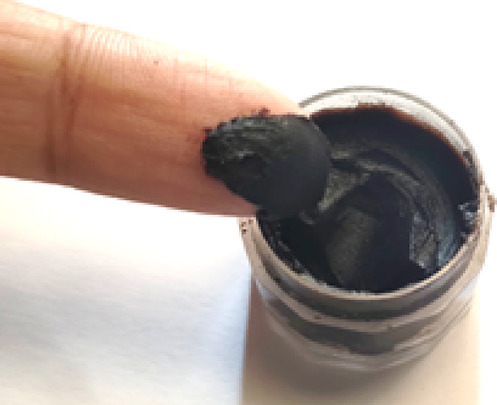
Commercially available propolis cream used in the experimental treatment.

The propolis cream used in this study (Fig. 1) was procured from a specialized bee extract store in Port Said city, Egypt. Propolis is a natural resinous substance collected by bees from various plants, known for its complex mixture of bioactive compounds. It exhibits potent anti-inflammatory and bactericidal properties, making it particularly effective in treating infectious wounds, burns, scalds, and accelerating wound healing. Previous studies have demonstrated its antioxidant capacity, which remains stable regardless of the concentration in ethanol solutions. Additionally, propolis formulated into liposomal preparations showed enhanced antioxidant activity compared to standard solutions, with some liposome formulations exhibiting significant bioactivity even without propolis content, likely due to inherent antioxidant properties of the delivery system itself^5–7^.

#### Preparation of *Persea americana* cream

**Fig. 2:**
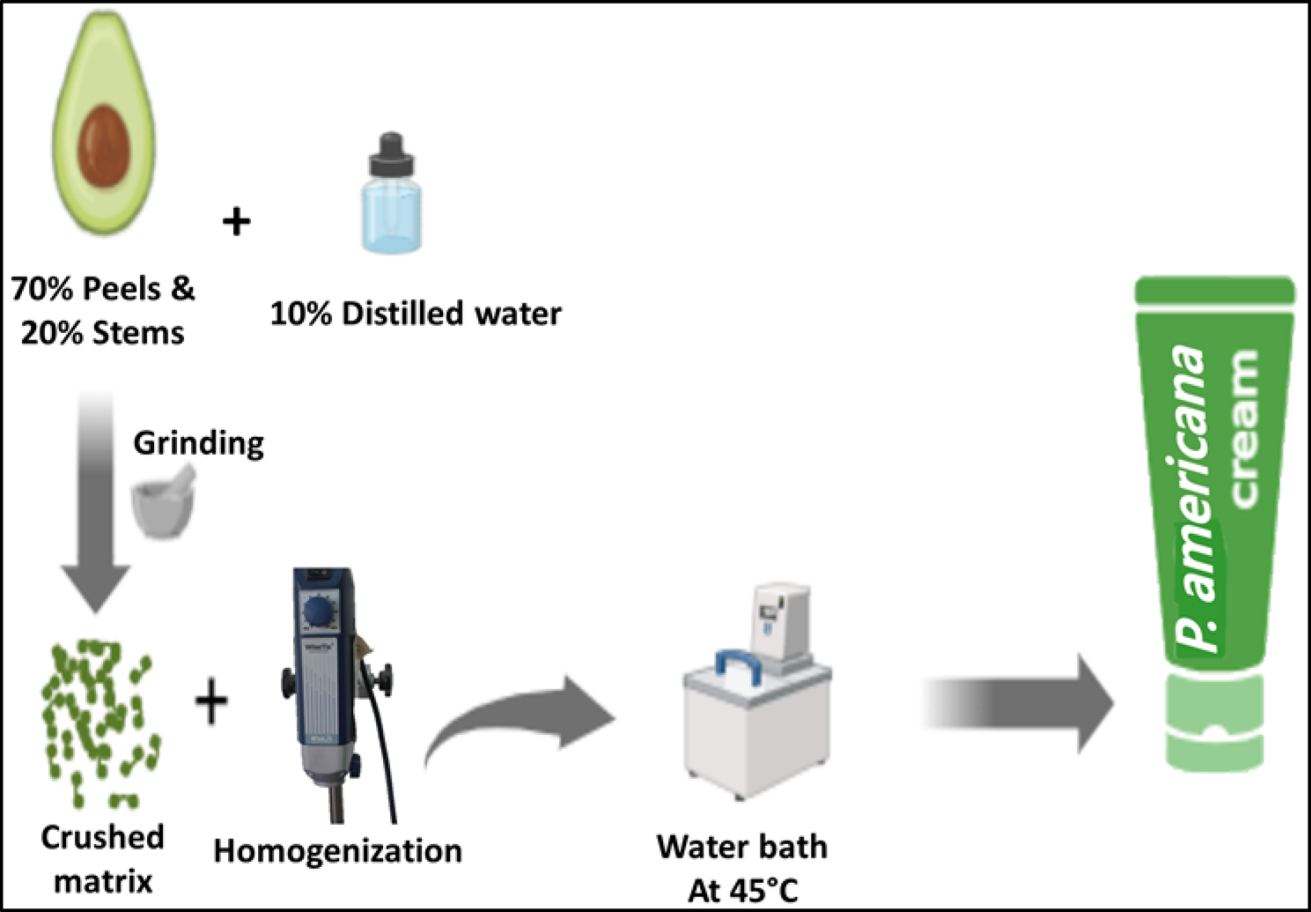
Step-by-step illustration of the preparation process of the *Persea americana* cream formulated from *Persea americana* peels and stems, including grinding, homogenization, and incubation in a water bath to obtain a uniform topical formulation.

Fresh peels (70%) and stems (20%) of *Persea Americana*^17^ were sourced from a commercial fruit store in Port Said city, Egypt. These plant materials were finely ground using a mortar grinding device (Retsch RM 200, Germany) to obtain uniformly small particles (~ 300 µm). Distilled water (10% of the total material weight) was then added to the crushed mixture, and the components were thoroughly blended using a WiseTis homogenizer (WiseTis HG-15A, Germany) to achieve a consistent and homogeneous mixture. This blend was subsequently placed in a water bath (NS BIOTEC, WB-30, Egypt) maintained at 45 °C for 3 h, with continuous shaking to ensure thorough integration of water with the plant material (see Fig. 2). The resulting uniform creamy formulation was designated as the *Persea americana* cream used in this study. The prepared *Persea americana* cream was aliquoted into 15 tubes and stored at 5 °C in a freezer throughout the experimental period.

### GC/MS analysis for bio-active compounds

Gas Chromatography Mass Spectrometry (GC/MS) analysis was conducted to identify and compare the bioactive constituents present in the commercial propolis cream and the formulated *Persea americana* cream, aiming to correlate compound profiles with their potential wound healing properties in rats. The analysis was performed using an ISQ 7000 GC–MS system. The GC component was equipped with a capillary column (30 m length × 250 µm internal diameter × 0.25 µm film thickness). Helium served as the carrier gas at a constant flow rate of 1 mL/min. The injector temperature was set at 260 °C. The oven temperature was programmed to increase in a stepwise manner: initially from 75 to 200 °C at a rate of 10 °C/min, followed by an increase to 280 °C at 5 °C/min, with a final isothermal hold at 280 °C for 15 min. Mass spectra were acquired using electron ionization (EI) mode at 70 eV, with a scan range of 20–620 Da and a scan interval of 0.5 s. Compound identification was accomplished by comparing the obtained mass spectra with entries in the NIST11 (National Institute of Standards and Technology) mass spectral database. The relative abundance of each compound was calculated based on the peak areas in the total ion chromatograms^3,18,19^.

### Animal experiment

Twelve healthy male albino rats, each weighing between 120 and 125 g, were used in this study. Upon arrival from the Veterinary Animal Center in Helwan City, Egypt, the animals were housed individually in clean, ventilated plastic cages under standard laboratory conditions. Environmental parameters, including temperature and humidity, were maintained within acceptable ranges, and good hygiene practices were strictly followed. The rats were provided with free access to clean water and a balanced diet containing essential nutrients, with feeding conducted once daily. A one-week acclimatization period was allowed prior to the commencement of the experiment to ensure proper adaptation to the new environment.

#### Body weight monitoring

The body weights of all experimental rats were monitored daily using a high-precision digital balance (accuracy ± 0.01 g). Weighing was conducted at the same time each day to minimize diurnal fluctuations related to feeding and metabolic activity. Prior to the experimental procedures, all animals underwent a one-week acclimatization period under controlled laboratory conditions, with unrestricted access to water and a nutritionally balanced standard diet composed of carbohydrates, proteins, fats, fiber, and essential minerals. Throughout the experiment, weight gain was calculated for each animal to evaluate the general health status and any potential systemic effects of the applied formulations. Weight gain was determined using the following equation^20^:

$${\text{The}}\;{\text{weight}}\;{\text{gain}}\left( {\text{g}} \right) = \frac{{{\text{Weight}}\left( {{\text{Cw}}} \right) - {\text{Weight}}\left( {{\text{Lw}}} \right)}}{{{\text{Weight}}\left( {{\text{Lw}}} \right)}}$$

where: Weight(Cw): The body weight of the rat in the current week, Weight(Lw): The body weight of the rat in the preceding week.

The resulting data were analyzed to assess temporal changes and evaluate the influence of treatment conditions over the course of the experiment.

#### Wound creation and treatment protocol

Prior to wound induction, the rats were anesthetized using intraperitoneal injection of ketamine (50 mg/kg) and xylazine (5 mg/kg), as recommended in previous protocols^21^. Dorsal hair was carefully shaved using an electric razor^5^, and the skin was cleaned with 70% ethanol. A full-thickness excisional wound measuring approximately 1 cm in diameter was then created on the dorsum of each rat using a sterile disposable surgical blade (size 10) to ensure standardized wound margins. Gentle dissection with surgical scissors was performed to separate the skin from the underlying tissue^3^.

**Fig. 3:**
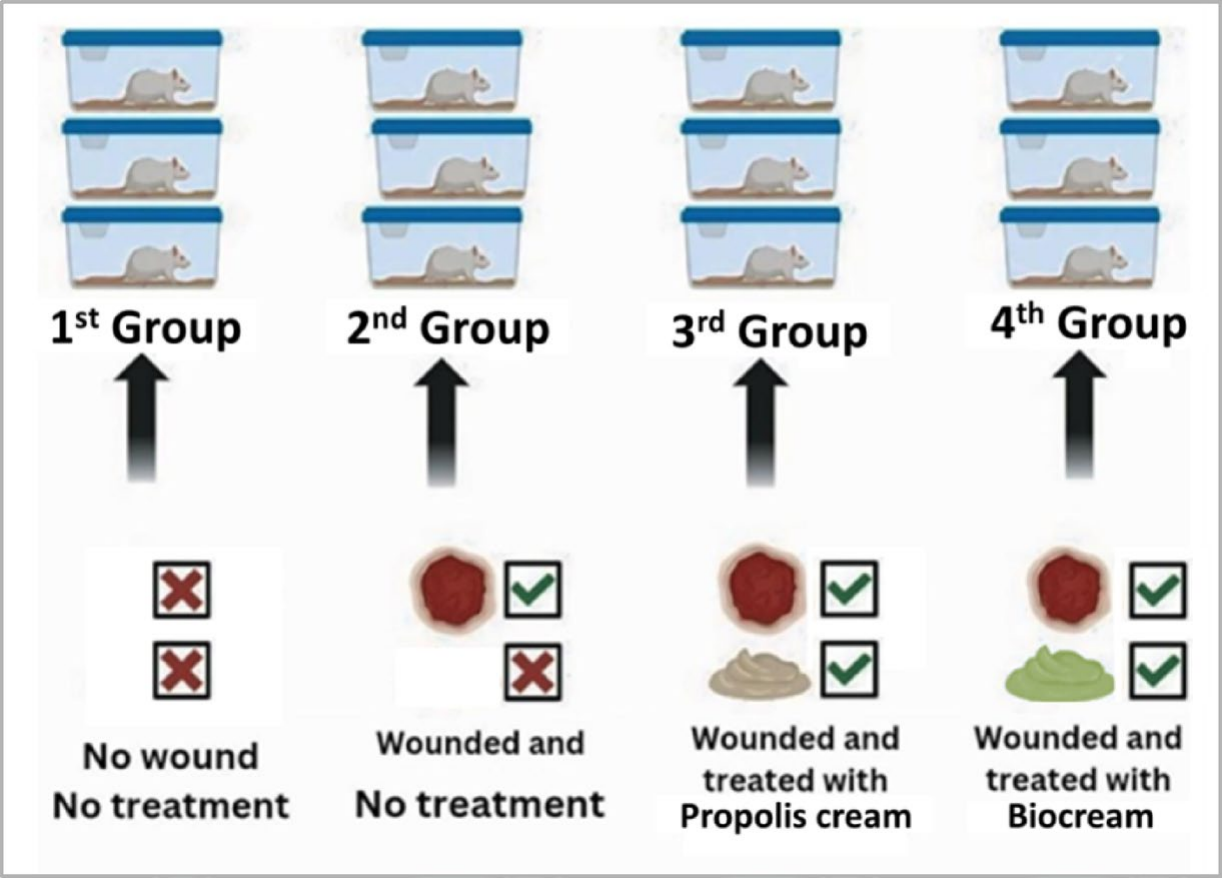
Overview of the experimental animal groups:1st Group (negative control), 2nd Group (positive control with untreated wounds), 3rd Group (wound treated with propolis cream), and 4th Group (wound treated with *Persea americana* cream).

The animals were randomly divided into four groups (n = 3 rats per group) as follows (Fig. 3):

- 1st Group (Negative Control): No wound induction.
- 2nd Group (Positive Control): Wounded but left untreated.
- 3rd Group (Propolis-Treated): Wounded and treated topically with 0.1 g/cm^2^ of commercial propolis cream once daily^22^.
- 4th Group (*Persea americana* cream Treated): Wounded and treated topically with 0.1 g/cm^2^ of *Persea americana* cream once daily^17^.

For all wounded groups (2nd, 3rd, and 4th Groups), the wound site was sterilized with Betadine prior to treatment application. The treatment lasted for 15 consecutive days, during which the wounds were visually monitored, and their surface area was measured. The percentage of wound contraction was calculated using the following formula^23–26^:

$${\text{Contraction}}\;{\text{of}}\;{\text{wound}}\;{\text{area}}\left( \% \right) = \frac{{{\text{W}}\left( 0 \right) - {\text{W}}\left( {\text{x}} \right)}}{{{\text{W}}\left( 0 \right)}} \times { }100$$

Where W(0): Initial wound area (on Day 0), W(x): Wound area on the day of assessment (Day x).

#### Biochemical and hematological assessments

At the end of the experimental period, all rats were fasted for 24 h and then anesthetized using an intraperitoneal injection of ketamine (50 mg/kg) combined with xylazine (5 mg/kg)^21^. While under deep anesthesia, blood samples were collected from the saphenous vein^27^. Approximately 1.5–2.0 mL of blood was collected per animal, sufficient for all hematological and biochemical assays. Following blood collection, euthanasia was performed via cervical dislocation using a sharp surgical blade, ensuring rapid and humane termination.

Whole blood samples were centrifuged at 3000 rpm for 10 min at 4 °C to ensure proper serum separation without degradation of labile components to separate plasma, which was used for the assessment of coagulation parameters. Prothrombin time (PT) and partial thromboplastin time (PTT) were measured using a CoData 504 analyzer (Siemens, Germany), employing standard PT and PTT reagents provided by the manufacturer.

For serum biochemical analysis, samples were allowed to clot, then centrifuged, and the supernatant serum was collected. Serum albumin was determined using a colorimetric assay kit (Biosystems, Spain), while glucose concentration was measured with a commercial kit (Agappe Diagnostics, India). Both were analyzed on a fully automated chemistry analyzer (Mindray BC-2800Vet, Mindray Bio-Medical Electronics Co., Ltd., China), and calibration was conducted prior to use. Blood samples were collected in EDTA-coated tubes and analyzed within 30 min to ensure accuracy and sample integrity.

Hemoglobin levels were measured using the Sahli’s method. Complete blood count (CBC), including white blood cell (WBC) count, red blood cell (RBC) count, and hematocrit (HCT), was performed on whole blood using an automated hematology analyzer (Mindray BS-600 m). The mean corpuscular volume (MCV) was calculated using the formula^28^: MCV = Fl/Cell. All measurements were conducted under standardized laboratory conditions, and proper sample handling protocols were followed to ensure data reliability and reproducibility.

#### Histopathological study

After completing the experimental period, skin tissue samples were collected and immediately fixed in 10% neutral buffered formalin for 48 h. Following fixation, tissues were dehydrated through a graded series of ethyl alcohol concentrations, cleared with xylene, and then infiltrated and embedded in paraffin wax to prepare tissue blocks. Sections were cut using a rotary microtome (RM2125, Leica, Germany) and mounted onto glass slides. The sections were then dewaxed and rehydrated through a descending alcohol series. Staining was performed using hematoxylin and eosin (H&E) as well as Masson’s trichrome (MT) stains. Finally, the stained slides were examined under a light microscope (BX51TF, Olympus, Japan) for histopathological evaluation^29–31^.

#### Statistical analysis

All statistical analyses were carried out using SPSS software version 21. Results are presented as mean values ± standard error (M ± SE). Before conducting ANOVA, the normality of data distribution was assessed using the Shapiro–Wilk test to ensure the appropriateness of parametric tests. One-way ANOVA was then applied to compare differences between experimental groups. Further pairwise comparisons were performed using Tukey’s and Duncan’s post-hoc tests to identify specific group differences. Statistical significance was set at *P* < 0.05, while *P* < 0.01 was considered highly significant. Data visualization through graphs and tables was used to clearly illustrate the experimental outcomes^21,32^.

### Ethical approval

All experimental procedures involving animals were conducted in accordance with the guidelines for the care and use of experimental animals approved by the Faculty of Science Committee at Port Said University, Port Said, Egypt (Approval No: ERN: PSU Sci. 101).

## Results

### The GC/MS analysis of commercial propolis cream

**Fig. 4:**
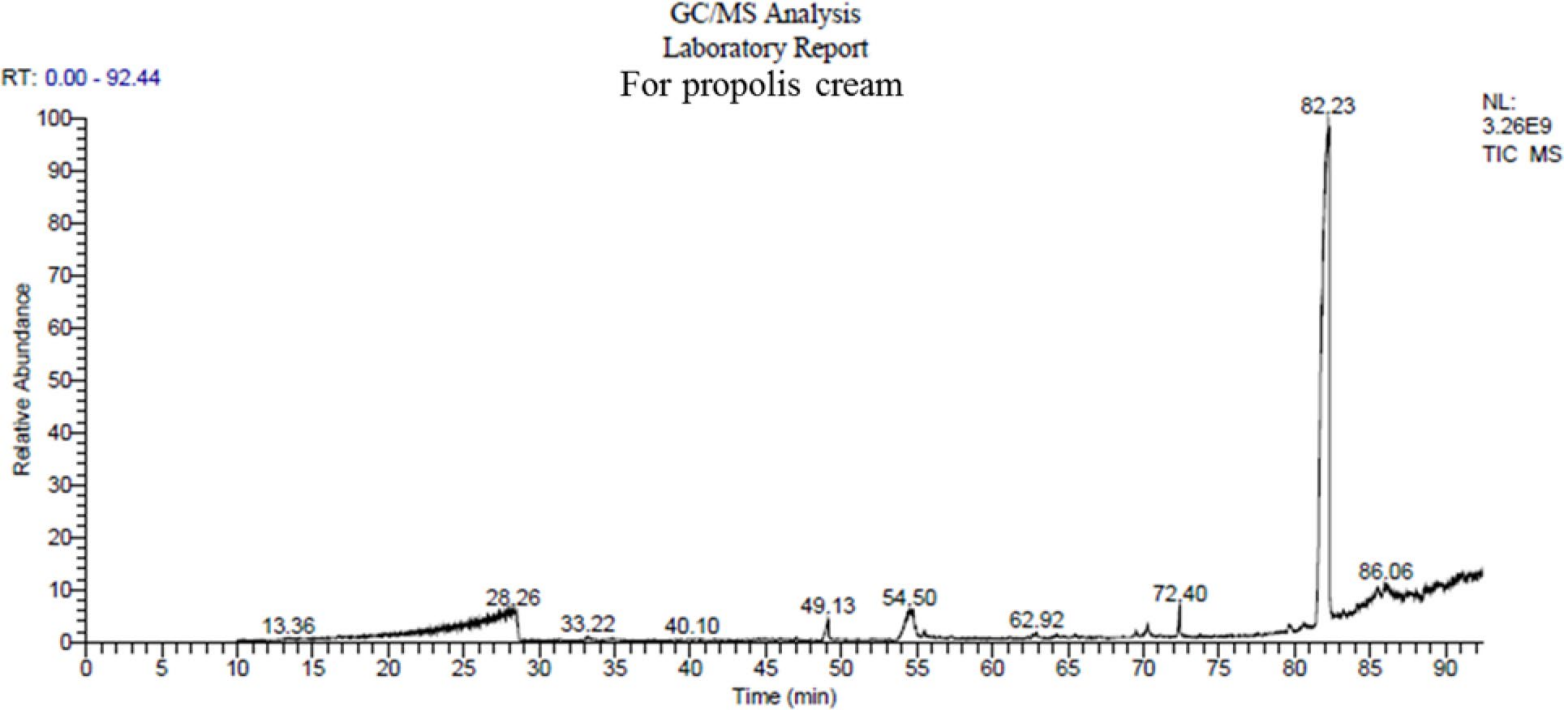
Representative GC/MS chromatogram of the commercial propolis cream, showing the separation of bioactive compounds identified based on their retention times and mass spectral profiles.

The chemical composition of the commercial propolis cream was characterized using Gas Chromatography-Mass Spectrometry (GC/MS). This analysis revealed the presence of various bioactive compounds. The key constituents were identified by their retention times (RT, in minutes) and relative peak areas (%), as detailed in Fig. 4 and summarized in Table 1. Notably, several of the identified compounds are associated with biological activities relevant to wound healing, such as antimicrobial, antioxidant, and anti-inflammatory effects. These include flavonoid derivatives and phenolic compounds, which may collectively contribute to the observed therapeutic potential of propolis in skin regeneration. The presence of these bioactive molecules supports the rationale for its topical application and offers a mechanistic explanation for its performance in the experimental wound mode.

**Table 1:** Identified chemical compounds in the commercial propolis cream based on GC/MS analysis, including retention times and relative peak areas (%), indicating the abundance of each bioactive component:

Retention time (min.)	Separated compounds	Molecular weight	Peak area (%)	Library
28.47		88	1.75	nist_ms ms
49.12		256	0.75	WileyRegistry8e, replib
54.50		280	1.20	WileyRegistry8e, replib
72.40		337	0.99	mainlib, replib
82.24		472	83.90	replib, mainlib, WileyRegistry8e

### The GC/MS analysis of *Persea americana* cream formulation

**Fig. 5:**
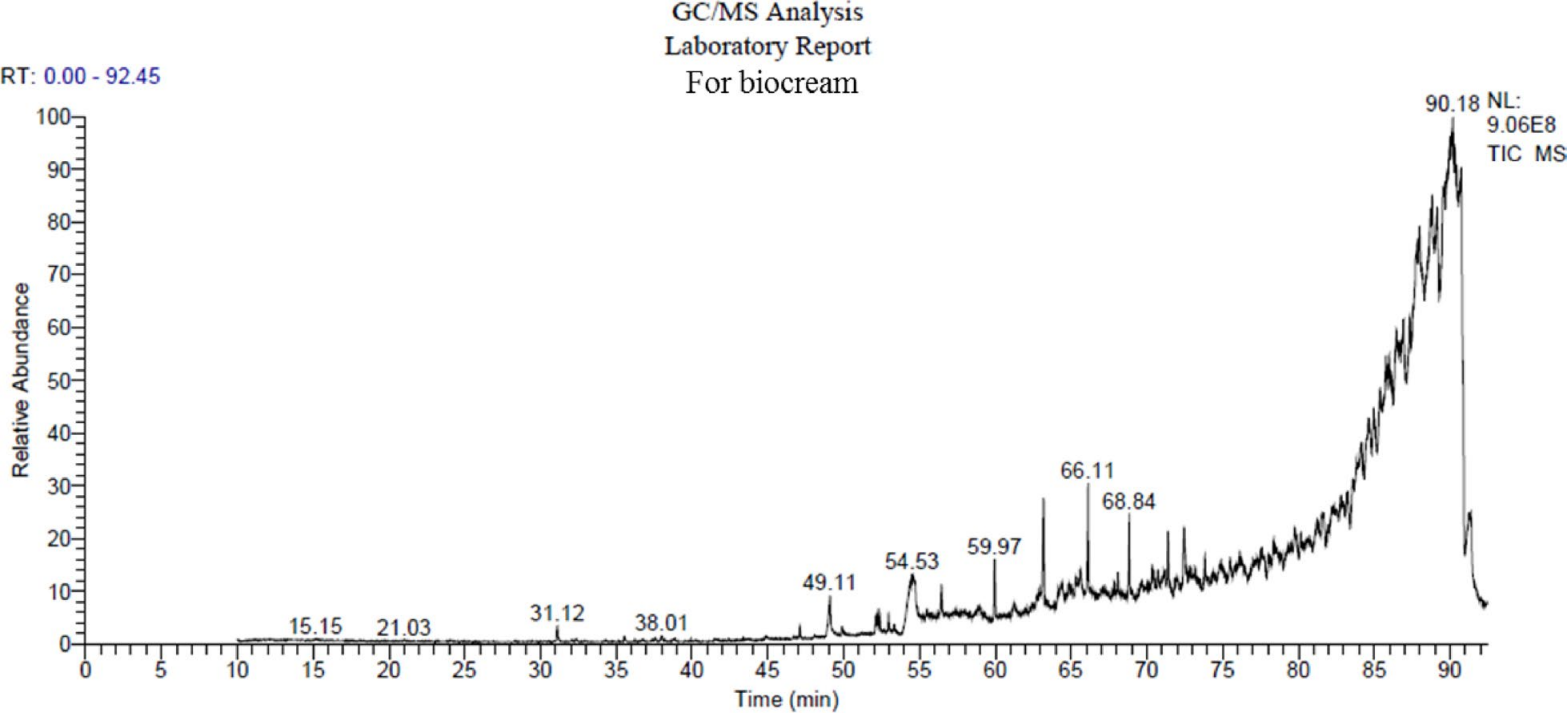
Gas Chromatography Mass Spectrometry (GC/MS) chromatogram depicting the bioactive compound profile of the *Persea americana* cream formulation.

Gas Chromatography-Mass Spectrometry (GC/MS) analysis of the *Persea americana* cream revealed a diverse spectrum of bioactive compounds. The major constituents were identified based on their retention times (RT, in minutes) and relative peak areas (%), indicating their abundance within the formulation, as shown in Fig. 5 and detailed in Table 2. Many of these compounds exhibit well-documented biological activities such as antimicrobial, anti-inflammatory, and antioxidant effects. These properties likely act in a synergistic manner to enhance the wound healing efficacy observed in our experimental model. The richness and diversity of bioactive components in the *Persea americana* cream may explain its superior therapeutic performance compared to the commercial propolis cream, supporting its potential as an effective natural agent for epidermal biorestoration.

**Table 2:** The identification of chemical compounds in the *Persea americana* cream formulation based on Gas Chromatography Mass Spectrometry (GC/MS) analysis.

Retention time	Separated compounds	Molecular weight	Area (%)	Library
31.12		220	0.47	mainlib
49.11		256	1.15	replib, mainlib, WileyRegistry8e
52.11		294	0.49	WileyRegistry8e, mainlib
52.23		292	0.60	WileyRegistry8e
52.40		296	0.49	WileyRegistry8e, replib
52.95		310	0.49	WileyRegistry8e
54.39		280	1.78	WileyRegistry8e, replib
54.63		282	1.21	WileyRegistry8e
63.19		492	2.79	WileyRegistry8e
64.30		298	0.53	replib
65.30		592	0.39	WileyRegistry8e, mainlib
65.62		310	0.70	mainlib
66.12		380	2.99	WileyRegistry8e, replib
67.84		490	0.38	mainlib
70.72		450	0.59	WileyRegistry8e
71.14		346	0.70	WileyRegistry8e, mainlib
71.97		504	0.97	WileyRegistry8e, mainlib
72.45		338	1.95	replib
73.18		914	0.46	WileyRegistry8e
78.35		532	0.66	WileyRegistry8e, replib
81.21		310	1.31	mainlib
81.96		588	0.47	mainlib
85.99		616	1.21	mainlib
89.52		560	2.10	mainlib

### Weight gain

**Fig. 6:**
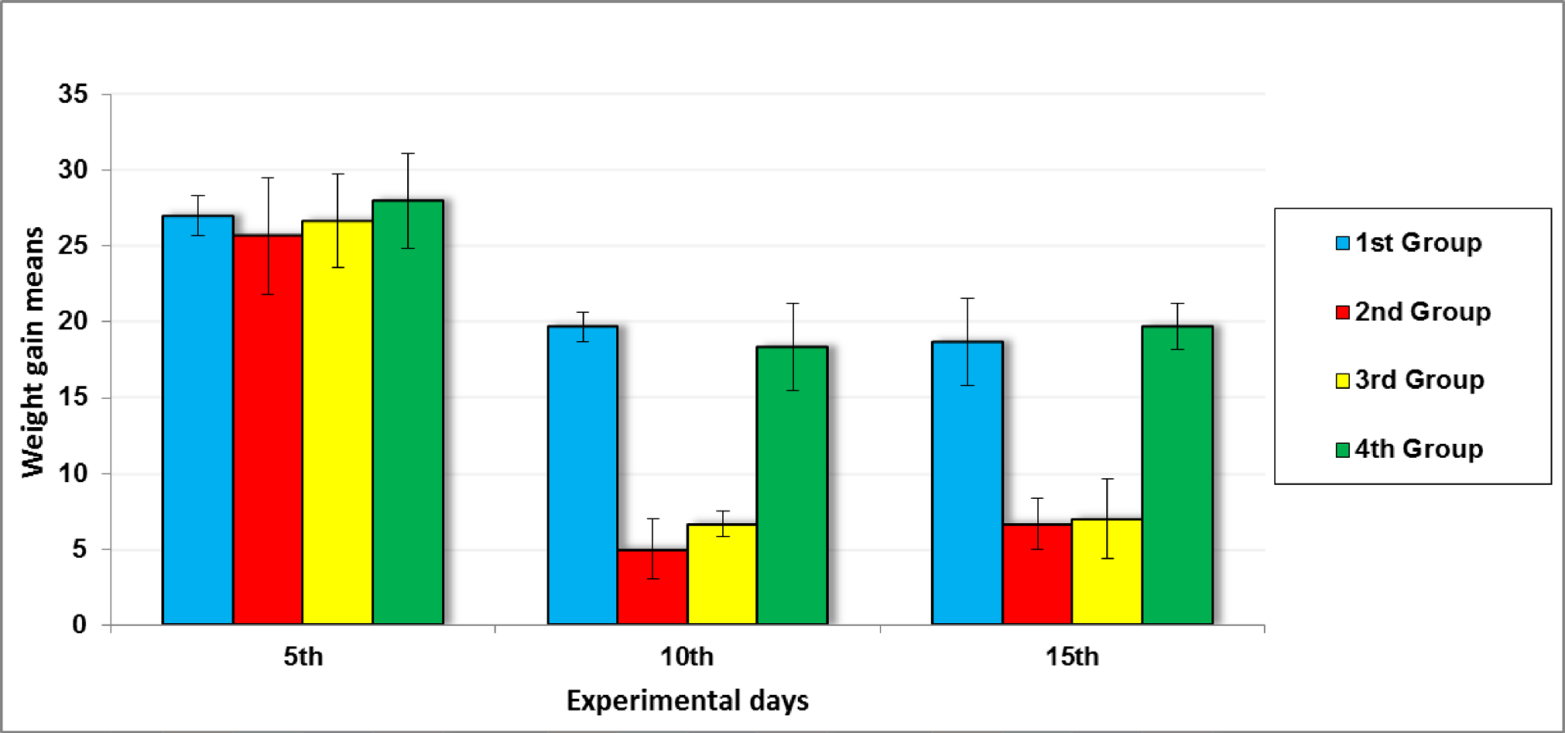
Mean weight gain of experimental groups throughout the study. 2nd and 3rd Groups showed a decrease in weight gain, whereas 1st and 4th Groups exhibited an increase.

As illustrated in Fig. 6, all four groups exhibited comparable weight gain ranging between 25 and 30 g by day 5 of the experiment. However, by day 10, a notable reduction in weight gain was observed in 2nd Group (positive control) and 3rd Group (propolis-treated) compared to 1st Groups (negative control) and 4th Group (*Persea americana* cream treated). This trend persisted until the end of the study period on day 15, where 2nd and 3rd Groups continued to show significantly lower weight gain relative to 1st and 4th Groups. These findings suggest that treatment with the *Persea americana* cream may better support overall health and weight maintenance during wound healing compared to propolis treatment or no treatment.

### Wound contraction in rats

**Fig. 7:**
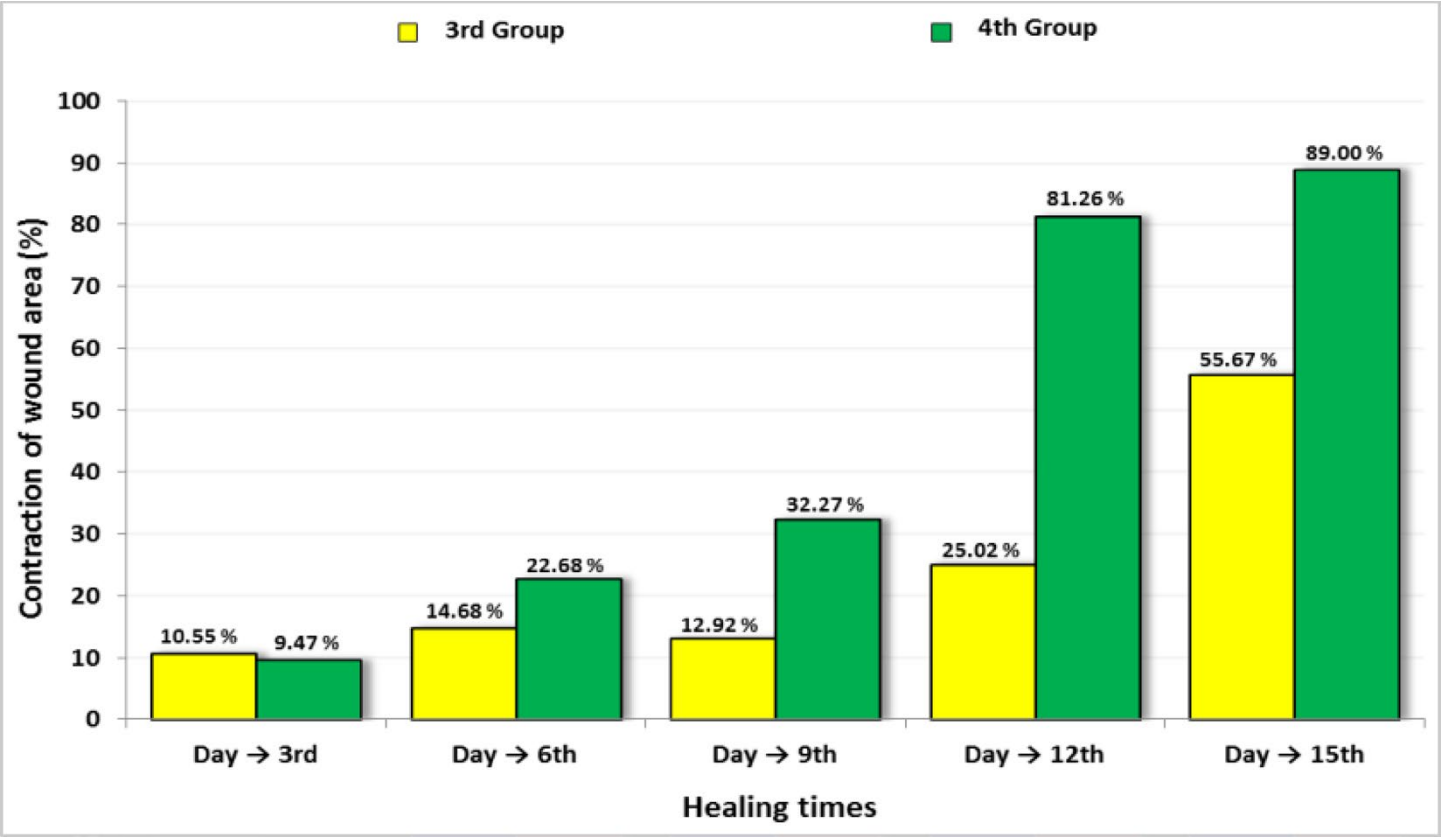
Comparison of wound contraction percentage between rats treated with propolis cream (3rd Group) and those treated with *Persea americana* cream (4th Group) over the 15-day experimental period.

Throughout the 15-day experimental period, the effect of topical application of propolis cream and *Persea americana* cream on wound closure was assessed by measuring wound diameter at regular intervals. While all groups exhibited progressive wound contraction, the rate and extent of contraction varied noticeably among treatments (Fig. 7). By day 6, a significant reduction in wound size was observed in rats treated with the *Persea americana* cream compared to both the untreated positive control and the group receiving propolis. This trend continued through days 9 and 12, with the *Persea americana* cream group showing more rapid wound closure, reaching an average wound diameter of 2.00 ± 1.15 mm on day 12, in contrast to 8.00 ± 1.01 mm in the propolis group and 10.67 ± 1.33 mm in the untreated group. By the end of the study (day 15), the *Persea americana* cream group achieved near-complete contraction, with wound diameter reduced to 0.33 ± 0.10 mm, significantly outperforming the propolis-treated group (1.33 ± 0.67 mm) and the untreated group (3.00 ± 0.58 mm). The enhanced wound contraction potential of the *Persea americana*-based formulation relative to both the propolis treatment and no treatment.

**Fig. 8:**
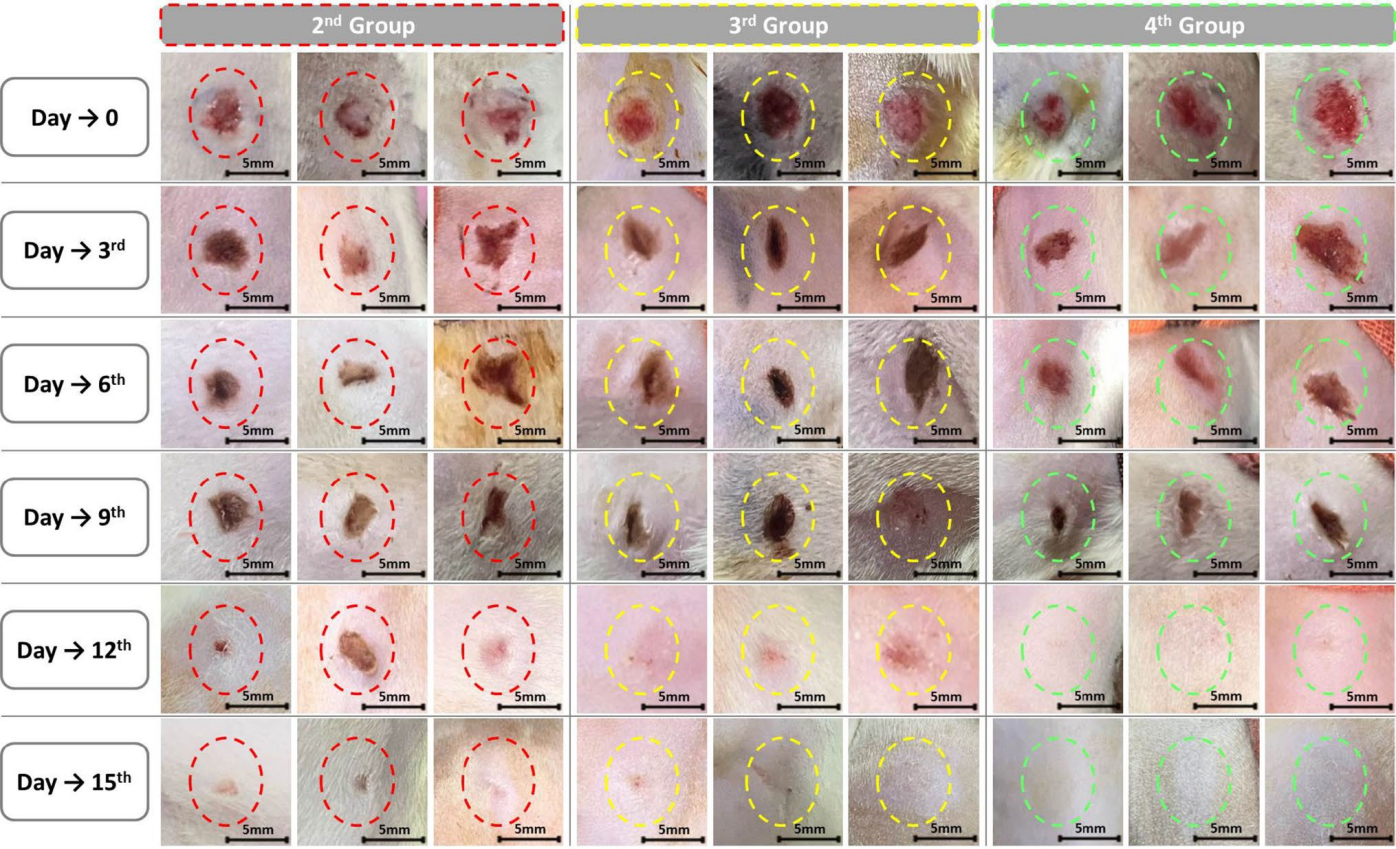
Macroscopic evaluation of wound healing progression over 15 days in the experimental group: 2nd Group (untreated), 3rd Group (treated with propolis cream), and 4th Group (treated with *Persea americana* cream). Progressive wound closure is visually evident, with faster healing observed in the 4th Group. A 5 mm scale bar is included to indicate the approximate wound size at each time point.

**Fig. 9:**
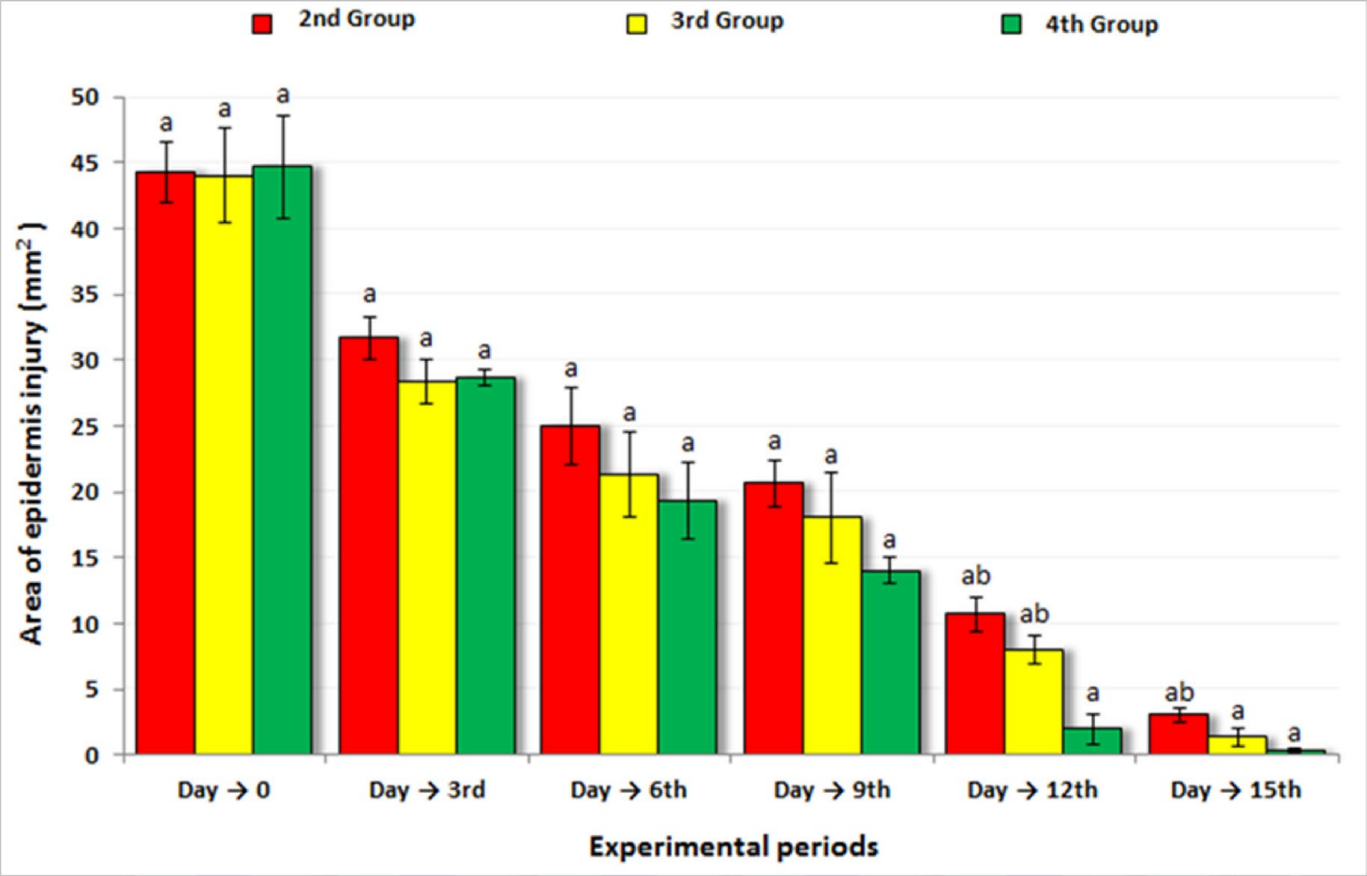
Changes in epidermal wound area (mm^2^) over the course of the experiment in the three injured groups: untreated control (2nd Group), propolis-treated (3rd Group), and *Persea americana* cream-treated (4th Group).

**Fig. 10:**
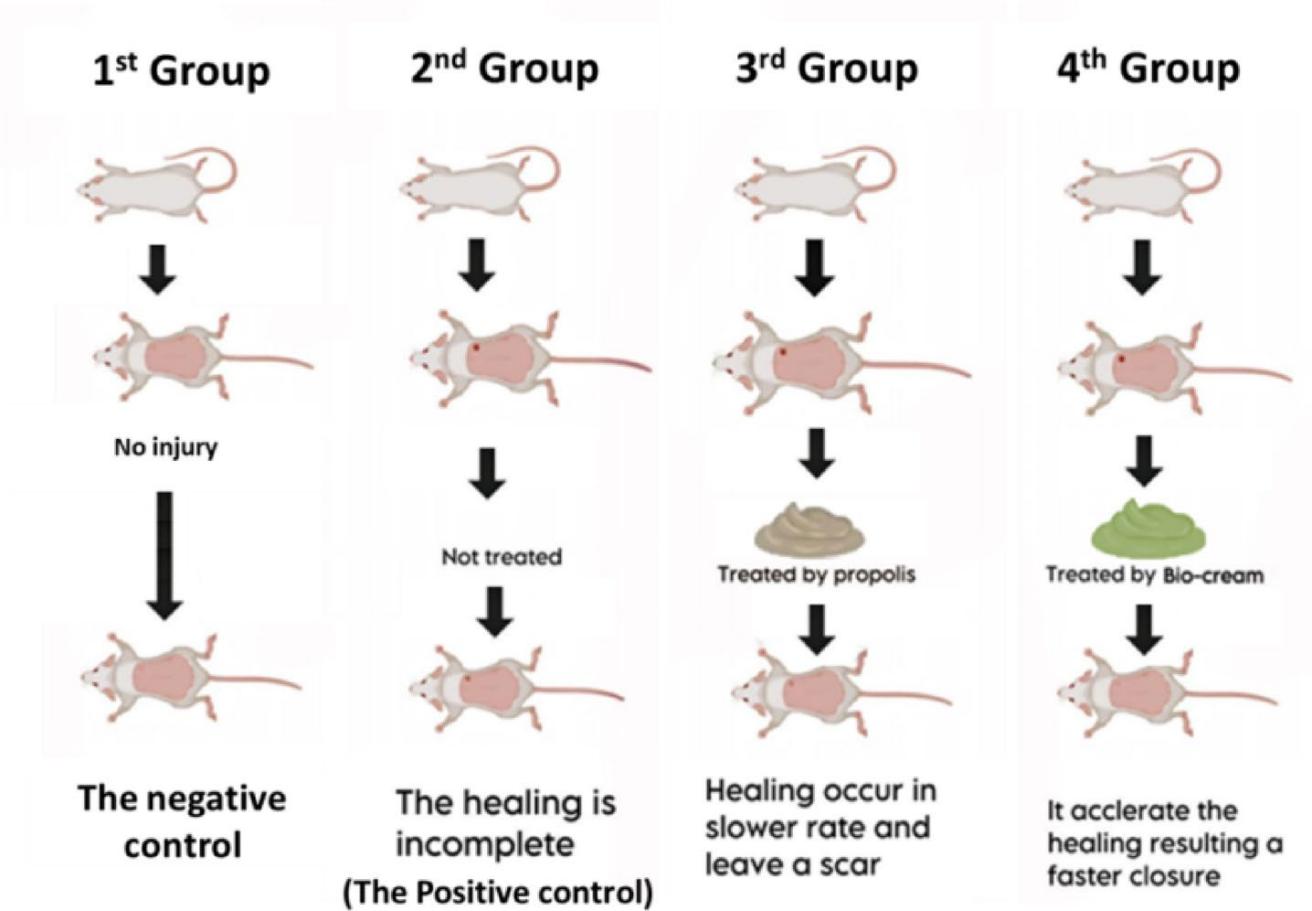
Comparative overview illustrating the impact of each treatment on wound closure dynamics and the extent of scar tissue formation after 15 days.

The Wound healing progression across the treated groups is illustrated in Figs. 8, 9, and 10. By the end of the 15-day experimental period, complete wound closure was achieved in all groups. However, a noticeably faster healing response was observed in the group treated with *Persea americana* cream (4th Group) compared to the propolis-treated group (3rd Group), indicating enhanced tissue regeneration associated with the *Persea americana* cream formulation.

### Physiological parameters:

#### Hematological parameters:

**Fig. 11:**
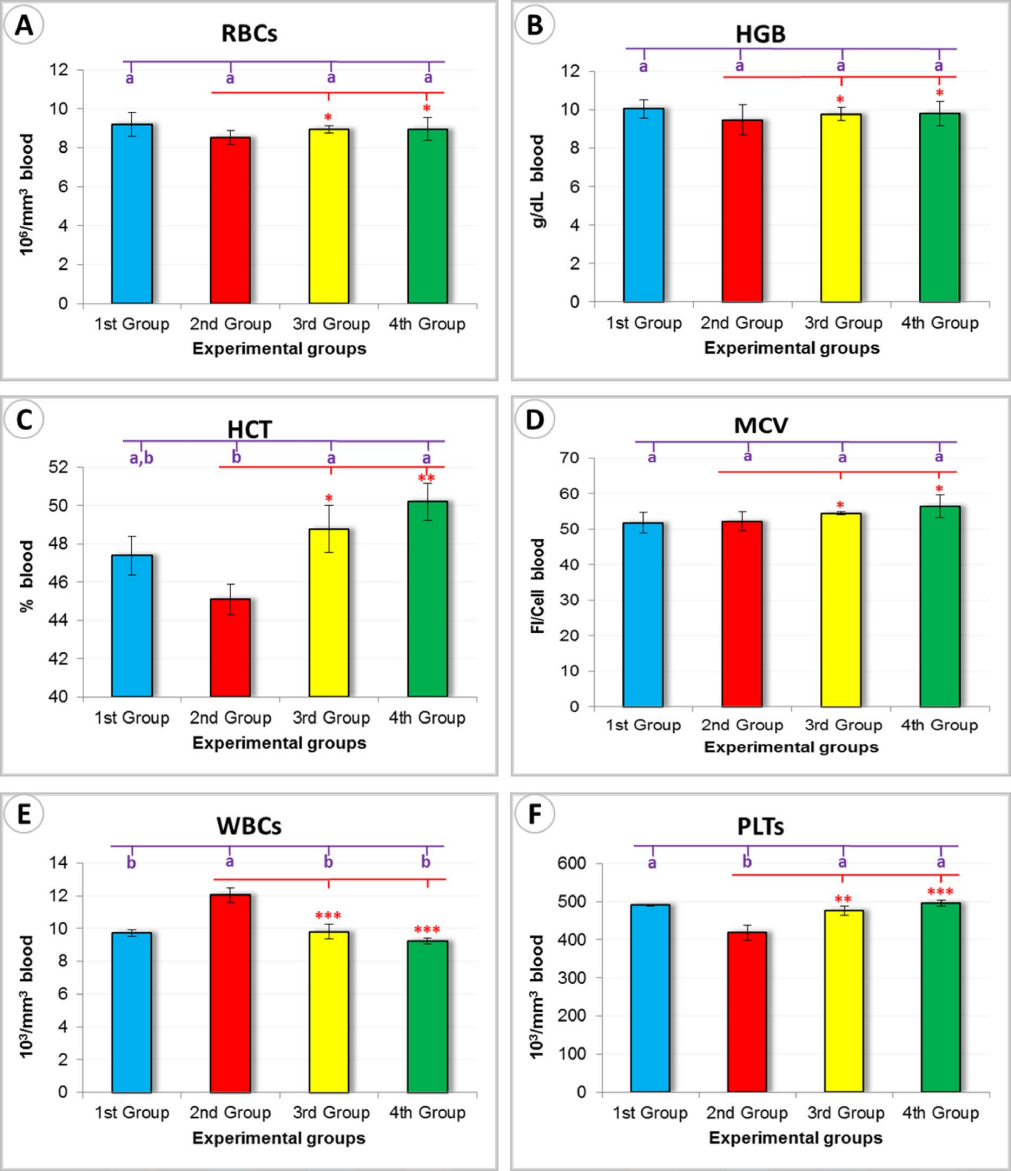
Graphical representation of hematological parameters measured across experimental groups: (**A**) Red Blood Cell count (RBCs), (**B**) Hemoglobin level (HGB), (**C**) Hematocrit percentage (HCT), (**D**) Mean Corpuscular Volume (MCV), (**E**) White Blood Cell count (WBCs), and (**F**) Platelet count (PLTs). Different superscript letters (a, b) indicate statistically significant differences between groups based on Duncan’s post hoc test. Statistical significance was also assessed using Tukey’s test, where *** ➔ *P* < 0.01, ** ➔ *P* < 0.05, and * ➔ *P* > 0.5.

Red Blood Cell (RBC) Count (million/mm^3^): Among all groups, the *Persea americana* cream-treated group (4th Group) recorded the lowest RBC count (8.95 ± 0.59), followed by the negative control group (1st Group) with (9.21 ± 0.60). The untreated wounded group (2nd Group) and the propolis-treated group (3rd Group) demonstrated slightly lower values (8.53 ± 0.36 and 8.94 ± 0.19, respectively). These differences were not statistically significant (*P* > 0.05), suggesting a modest impact on erythropoiesis across treatment groups (Fig. 11A).

Hemoglobin (HGB) (g/dL): The highest hemoglobin concentration was observed in the 1st Group (10.05 ± 0.48), while the remaining groups showed slightly reduced values: 2nd Group (9.47 ± 0.78), 3rd Group (9.77 ± 0.34), and 4th Group (9.79 ± 0.63). Although the differences were minor, the relatively stable HGB levels in the 4th Group may reflect a preserved oxygen-carrying capacity (Fig. 11B).

Hematocrit (HCT) (%): Statistical analysis revealed a significantly higher hematocrit level in the 4th Group (50.19 ± 0.98) compared to the other groups (*P* < 0.05), indicating an improved red blood cell mass in response to *Persea americana* cream treatment (Fig. 11C).

Mean Corpuscular Volume (MCV) (fL/Cell): The MCV values were markedly elevated in the 4th Group (56.47 ± 3.14), suggesting an increase in red cell volume. In contrast, the 1st and 2nd Groups showed relatively lower values (51.83 ± 2.97 and 52.19 ± 2.63, respectively), which may indicate a more active hematopoietic response in the *Persea americana* cream group (Fig. 11D).

White Blood Cell (WBC) Count (thousand/mm^3^): The 2nd Group displayed the highest WBC count (12.03 ± 0.46), followed by the 3rd and 4th Groups (9.80 ± 0.46 and 9.23 ± 0.18, respectively). These values were significantly different (*P* < 0.01), possibly reflecting the persistence of inflammation in the untreated group and a moderated immune response in the treated groups (Fig. 11E).

Platelet Count (PLTs) (thousand/mm^3^): A significant elevation in platelet count was detected in the 4th Group (495.67 ± 8.29), compared to the 2nd Group, which had the lowest count (419.00 ± 19.40) (*P* < 0.05 and *P* < 0.01). This finding suggests enhanced coagulation and wound-sealing potential in the *Persea americana* cream-treated group (Fig. 11F).

#### Coagulation parameters

**Fig. 12:**
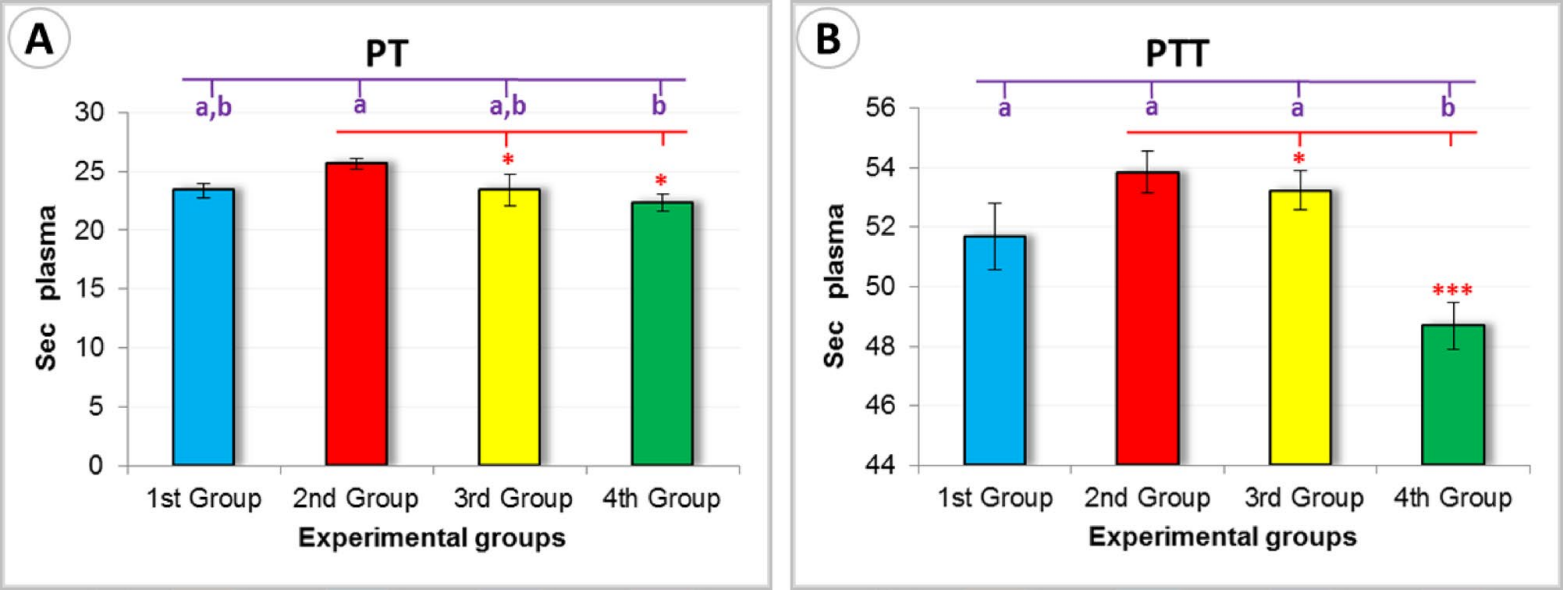
Graphical representation of coagulation parameters: (**A**) Prothrombin Time (PT) and (**B**) Partial Thromboplastin Time (PTT) across the experimental groups, illustrating the impact of different treatments. Different letters (**a**, **b**) indicate statistically significant differences based on Duncan’s test. Significance levels by Tukey’s test are indicated as follows: *** ➔ *P* < 0.01, ** ➔ *P* < 0.05, and * ➔  *P* > 0.5.

Analysis of coagulation parameters revealed that rats treated with the *Persea americana* cream formulation (4th Group) exhibited the shortest clotting times among all groups. Specifically, the 4th Group recorded the lowest prothrombin time (PT) at 22.31 ± 0.73 s (Fig. 12A), and the shortest partial thromboplastin time (PTT) at 48.68 ± 0.77 s (Fig. 12B). These findings suggest a more favorable coagulation profile, potentially supporting faster wound closure and hemostasis.

#### Biochemical parameters

**Fig. 13:**
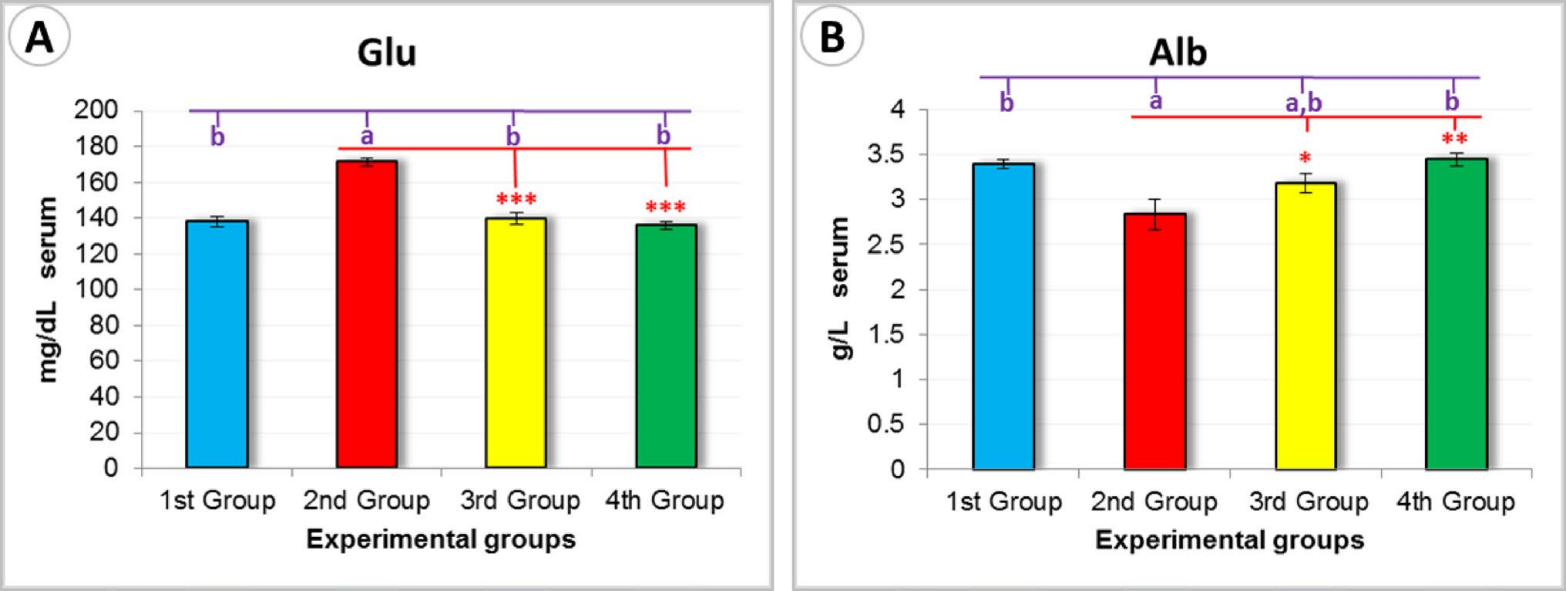
Graphical representation of glucose (Glu) and albumin (Alb) levels demonstrating the effects of the tested treatments. Different letters (**A**, **B**) indicate statistically significant differences according to Duncan’s test. Significance levels are also denoted as follows: *** ➔ *P* < 0.01, ** ➔ *P* < 0.05, and * ➔ *P* > 0.5, based on Tukey’s test.

Glucose (Glu) (mg/dl): The 2nd Group exhibited the highest glucose concentration (171.33 ± 2.33 mg/dL) compared to all other groups. In contrast, the 4th Group maintained a stable and significantly lower glucose level (136.00 ± 2.31 mg/dL), indicating better glycemic control during the healing process (*P* < 0.01) (Fig. 13A).

Albumin (Alb) (g/dL): The 4th Group demonstrated the highest serum albumin concentration (3.45 ± 0.07 g/dL), reflecting enhanced protein synthesis and improved nutritional status, which are crucial for effective wound healing (*P* < 0.05) (Fig. 13B).

### Histopathology findings

#### Skin sections stained with hematoxylin & eosin (H&E)

**Fig. 14:**
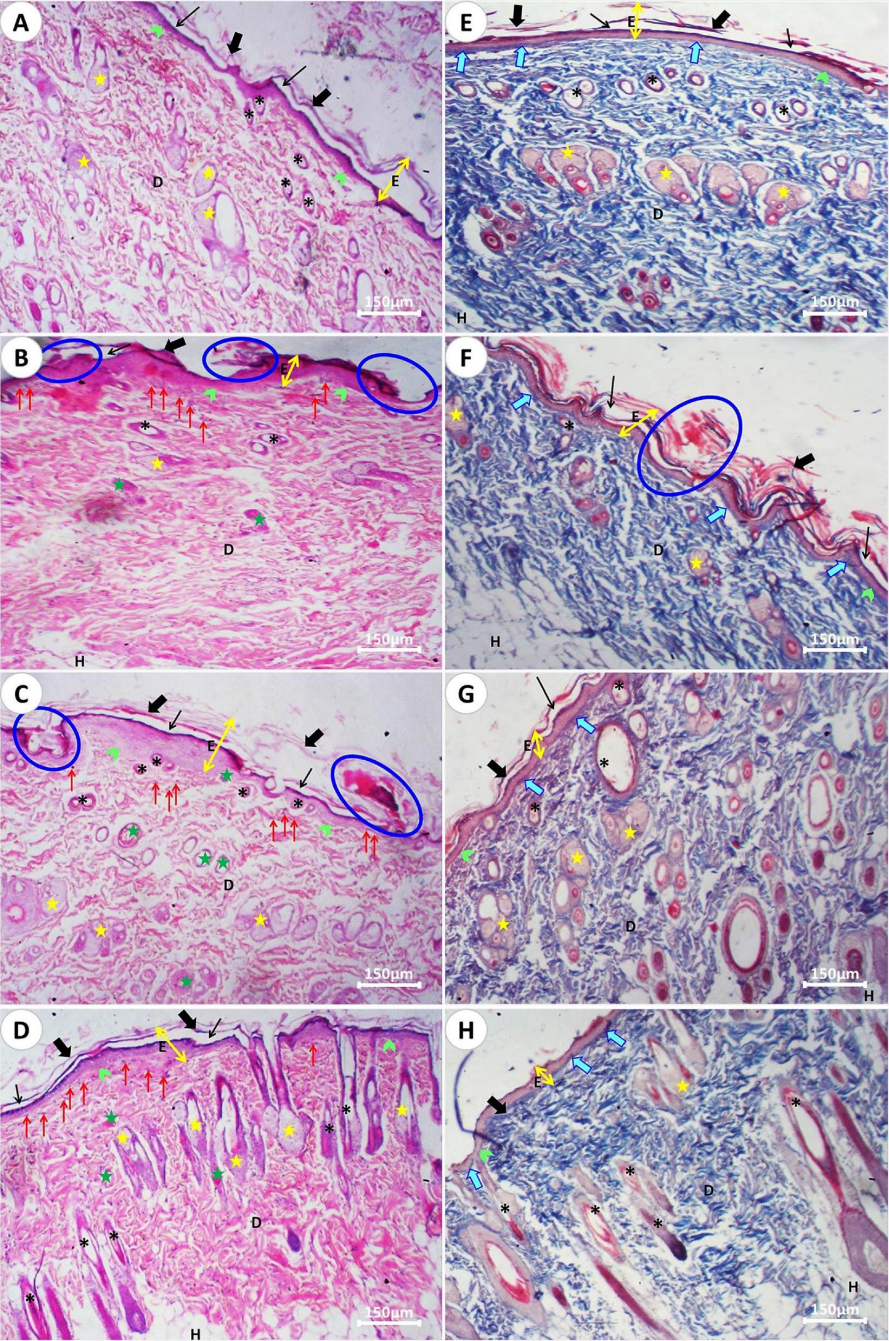
Photomicrographs of skin sections from the four experimental groups: Panels **A** and **E **represent the (1st Group), **B** and **F** the (2nd Group), **C** and **G** the (3rd Group), and **D** and **H** the (4th Group). Labels indicate: E ( ↔): epidermis, D: dermis, H: hypodermis; Black thick arrows: stratum corneum layer; Black thin arrows: stratum lucidum; Red thick arrows: inflammatory cells; Light blue arrows: collagen fibers; Green arrowhead: stratum basale; Blue circular frames: epidermal scratching and remaining scabs; Yellow stars: sebaceous glands; Green stars: blood vessels; Asterisks: hair follicles. Panels **A**–**D** are stained with Hematoxylin & Eosin (H&E), and panels **E**–**H** with Masson’s Trichrome (MT). Scale bar represents 150 µm.

The wound healing process in the experimental groups showed distinct histological features corresponding to the classical phases of inflammation, granulation tissue formation, contraction, and remodeling. In the control group (1st Group), the skin section displayed intact epidermal layers with a normal dermal architecture (Fig. 14A). In contrast, the 2nd Group (Fig. 14B), representing injured untreated skin, exhibited a pronounced inflammatory phase, characterized by marked erosion of the epidermal layers and infiltration of inflammatory cells, including neutrophils and Langerhans cells. This prolonged inflammation was accompanied by impaired granulation, reflected in the presence of weakly stained collagen fibers in the dermis.

Progression into the remodeling phase was noted, with keratinocyte proliferation leading to the formation of a new epidermal layer, reduced inflammatory infiltrate, enhanced angiogenesis, and gradual restoration of collagen fibers with normalized pink staining. These regenerative changes were most prominent in the 4th Group (Fig. 14D), where both epidermal and dermal layers displayed near-normal architecture. Moreover, this group showed a marked increase in glandular structures, hair follicles, and associated appendages, suggesting that *Persea americana* cream promoted hair regeneration and comprehensive tissue repair compared to the 2nd and 3rd Groups (Fig. 14B, C, respectively).

#### Skin sections stained with Masson’s trichrome (MT)

In the control group (1st Group), the skin section exhibited normal architecture with well-organized collagen fibers (Fig. 14E). In contrast, in the wounded skin of the 2nd Group (Fig. 14F), the healing process appeared incomplete. The presence of a persistent scab on the wound surface, coupled with insufficient collagen fiber deposition and an abundance of inflammatory cells, such as Langerhans cells and neutrophils, indicated a delayed transition from the inflammatory to the granulation phase. Sensory cells also appeared compromised, further supporting the observation of impaired healing.

Conversely, treatment with both creams accelerated wound healing by promoting the granulation and remodeling phases, as evidenced by increased accumulation of collagen fibers, which stained dark blue throughout the epidermal and dermal layers. This effect was most pronounced in the 4th Group (Fig. 14H), which demonstrated enhanced collagen synthesis and improved fiber organization compared to the 2nd and 3rd Groups (Fig. 14F, G, respectively). These findings highlight the superior capacity of *Persea americana* cream to stimulate collagen remodeling and tissue regeneration.

## Discussion

The current study highlights the distinct chemical profiles and biological efficacies of the *Persea americana* cream formulation, and the commercial propolis cream. GC/MS analysis revealed that the *Persea americana* cream contains a broader spectrum of bioactive compounds, including erucic acid, heptacosane, and dotriacontane, all known for their antimicrobial, anti-inflammatory, and immunomodulatory effects^10^. These compounds likely contribute synergistically to the enhanced wound healing observed in the *Persea americana* cream treated group, surpassing the effects of propolis cream which, although rich in flavonoids and phenolics, exhibited a comparatively narrower bioactive profile^33,34^.

Consistent with the chemical composition, in vivo results demonstrated that the *Persea americana* cream significantly accelerated wound contraction compared to both the untreated control and the propolis-treated groups. The *Persea americana* cream group showed the fastest and most complete wound closure by day 15, supporting the premise that its multi-faceted bioactive constituents promote more efficient tissue repair and regeneration^24–26,28^. This was further corroborated by histopathological findings, where skin sections from the *Persea americana* cream group displayed restored epidermal and dermal architecture, increased collagen fiber deposition, and enhanced angiogenesis, indicating superior remodeling of the extracellular matrix and reduction of inflammatory infiltrates^17^.

Hematological and biochemical parameters further substantiated these observations. The *Persea americana* cream group exhibited elevated hematocrit and mean corpuscular volume values, reflecting improved erythropoiesis and oxygen transport capacity, which are critical for tissue regeneration^35,36^. Meanwhile, white blood cell counts were lower in this group compared to the untreated controls, suggesting a resolved inflammatory phase conducive to healing. Notably, platelet counts were highest in the *Persea americana* cream group, indicating enhanced coagulation potential, which may facilitate early hemostasis at the wound site. In contrast, the propolis-treated group showed moderate improvements in these parameters, aligning with its established but less comprehensive biological effects^37,38^.

Furthermore, coagulation times (PT and PTT) were shorter in the propolis group, suggesting a role in accelerating clot formation; however, these effects were less pronounced in the *Persea americana* cream group, implying distinct mechanisms of wound healing enhancement between the two formulations. Biochemically, the *Persea americana* cream maintained stable glucose levels and increased serum albumin, supporting metabolic homeostasis and protein synthesis essential for tissue repair^37^.

Taken together, the findings highlight that the *Persea americana* cream formulation offers a comprehensive therapeutic benefit by combining antimicrobial, anti-inflammatory, and regenerative actions that synergistically enhance wound contraction. this broad-spectrum efficacy contrasts with the more limited, targeted effects observed with propolis cream. The study thus positions *Persea americana* cream as a promising candidate for topical wound management. Nevertheless, certain limitations should be acknowledged. The relatively small sample size and the 15-day observation period may reduce statistical power and limit the assessment of long-term outcomes, such as scar maturation and tensile strength recovery. These constraints, while common in rodent wound healing models^39,40^, do not undermine the value of the present results. The consistent improvements observed across histopathological, hematological, and wound contraction parameters reinforce the reliability of the findings. Further studies involving larger cohorts and extended follow-up periods are recommended to validate these outcomes and explore the underlying molecular mechanisms and potential clinical applications of this bioactive formulation.

## Conclusion

This study distinctly demonstrated the superior wound healing efficacy of the *Persea americana* cream compared to commercial propolis cream, as evidenced by comprehensive GC–MS profiling and significant improvements in hematological, biochemical, and histopathological parameters. The *Persea americana* cream not only accelerated wound closure but also effectively modulated inflammatory responses and enhanced tissue regeneration. These findings highlight the *Persea americana* cream’s multifaceted therapeutic potential, underscoring the value of its bioactive constituents. Future research is warranted to further elucidate its molecular mechanisms and optimize its application for clinical wound management, particularly focusing on collagen remodeling and inflammation control to maximize healing outcomes.

## Data Availability

All data regarding this study are presented in the paper.
